# Rothmund-Thomson syndrome, a disorder far from solved

**DOI:** 10.3389/fragi.2023.1296409

**Published:** 2023-11-10

**Authors:** Davi Jardim Martins, Ricardo Di Lazzaro Filho, Debora Romeo Bertola, Nícolas Carlos Hoch

**Affiliations:** ^1^ Genomic Stability Unit, Department of Biochemistry, Institute of Chemistry, University of São Paulo, São Paulo, Brazil; ^2^ Center for Human Genome Studies, Department of Genetics and Evolutionary Biology, Institute of Biosciences, University of São Paulo, São Paulo, Brazil; ^3^ Dasa Genômica/Genera, Genômica, São Paulo, Brazil; ^4^ Genetics Unit, Department of Pediatrics, Faculty of Medicine, Children’s Institute, Hospital das Clínicas, University of São Paulo, São Paulo, Brazil

**Keywords:** Rothmund-Thomson syndrome, RECQL4, ANAPC1, DNA2, CRIPT, poikiloderma

## Abstract

Rothmund-Thomson syndrome (RTS) is a rare autosomal recessive disorder characterized by a range of clinical symptoms, including poikiloderma, juvenile cataracts, short stature, sparse hair, eyebrows/eyelashes, nail dysplasia, and skeletal abnormalities. While classically associated with mutations in the *RECQL4* gene, which encodes a DNA helicase involved in DNA replication and repair, three additional genes have been recently identified in RTS: *ANAPC1*, encoding a subunit of the APC/C complex; *DNA2,* which encodes a nuclease/helicase involved in DNA repair; and *CRIPT*, encoding a poorly characterized protein implicated in excitatory synapse formation and splicing. Here, we review the clinical spectrum of RTS patients, analyze the genetic basis of the disease, and discuss molecular functions of the affected genes, drawing some novel genotype-phenotype correlations and proposing avenues for future studies into this enigmatic disorder.

## 1 Introduction

Genomic stability is the cellular ability to orchestrate DNA replication, DNA damage detection, lesion repair and cell cycle progression control, and thus ensuring that daughter cells receive a high-fidelity copy of the genome. Individuals that inherit genetic variants that cause one or more of these functions to become deregulated develop rare genetic disorders that are associated with increased genomic instability, cell death and/or cellular senescence, leading to premature aging, predisposition to cancer, neurodegeneration and/or developmental problems (Aguilera and Gómez-González, 2008). The study of these syndromes has been instrumental in shaping our understanding of the molecular function of the genes related to each of these diseases and the cellular processes they participate in. Thus, this article aims to review the Rothmund-Thomson syndrome (RTS—OMIM #618625; #268400), a genodermatosis in which poikiloderma is the main hallmark (Larizza et al., 2010). Despite being first described more than 150 years ago, the etiology of RTS and the physiopathology of its signs and symptoms are still far from solved.

### 1.1 Clinical characterization of Rothmund-Thomson syndrome

RTS was first described in 1868 by Auguste Rothmund who reported children from a Bavarian village presenting juvenile cataracts, short stature and skin eruptions (Rothmund, 1868). In 1923, Sydney Thomson described individuals with skin problems similar to those observed by Rothmund, but without juvenile cataracts and the presence of radial ray defects and short stature, naming the disease as “congenital poikiloderma” (Thomson, 1923). In 1957, Taylor postulated that these patients suffered from the same disease, coining the eponym Rothmund-Thomson syndrome (Taylor, 1957; Wang et al., 2001; Larizza et al., 2010).

The typical skin rash starts between 3 and 10 months of age as erythema on the face, associated with some swelling and, less often, blisters, spreads to extremities (extensor areas first, then flexor areas) and buttocks, sparing trunk and abdomen. In the evolution to a chronic phase, different types of lesions can be observed, such as telangiectasias, areas of reticulated hyperpigmentation and hypopigmentation and punctate atrophy, consistent with poikiloderma. In addition, one-third of patients develop hyperkeratosis, with excess accumulation of keratin in regions of the skin. The erythema can get worse after sun exposure. Other ectodermal findings include sparse/brittle/thin hair, sparse eyebrows/eyelashes; dystrophic nails; abnormal teeth (microdontia, abnormal crown formation, short roots, and increased incidence of caries) (Wang et al., 2001; Larizza et al., 2010; Wang and Plon, 2020). Another cardinal feature in RTS are cataracts, which are usually bilateral, juvenile and of rapid progression. Other ocular abnormalities include corneal anomalies, glaucoma, retinal atrophy, iris dysgenesis and strabismus. Individuals with RTS frequently present low birth weight and length that persist in clinical evolution, in some cases accompanied by growth hormone deficiency. Gastrointestinal anomalies are also seen, with feeding problems in infancy, sometimes requiring tube feeding; chronic diarrhea that resolves in early childhood and, occasionally, congenital anomalies, including anal atresia. Skeletal anomalies are characterized particularly by radial ray defects, but often also include patella hypoplasia/aplasia, osteopenia, irregular metaphysis with abnormal trabeculation and joint dislocations. Neurocognitive development is normal in the majority of patients (Wang et al., 2001; Larizza et al., 2010). Increased risk for cancer is a major concern in individuals with RTS, with an estimated prevalence of 30% for osteosarcoma and 5% for skin cancer (squamous cell carcinoma). Osteosarcoma, which could be isolated or multicentric, develops at a median age of 11.5 years, earlier than in non-syndromic cases. Metastatic osteosarcoma is one of the major causes of death in RTS patients, requiring careful clinical management. Other less frequently described tumors include malignant fibrous histiocytoma, basal cell carcinoma, Bowen´s disease, myelodysplasia, acute myeloid leukemia. This increased risk for cancer development is not observed in the heterozygous parents (Wang et al., 2001; Larizza et al., 2010; Wang and Plon, 2020).

## 2 Rothmund-Thomson syndrome diagnosis

While the clinical presentation of RTS patients can be heterogeneous and hamper a precise diagnosis (Haytaç et al., 2002; Stinco et al., 2008; Polese et al., 2011; Barisonek et al., 2016; Miranda et al., 2016; Pasagadugula et al., 2016; Chinmayee et al., 2017; Ahn et al., 2019; Zirn et al., 2021), current guidelines indicate that the individual must have, in addition to poikiloderma, at least two of the following clinical findings (Wang et al., 2001; Wang and Plon, 2020).

➢ Bilateral cataracts (usually juvenile);
➢ Dental abnormalities (defect in growth, enamel production or tooth eruption);
➢ Gastrointestinal disorder (chronic vomiting and diarrhea);
➢ Hyperkeratosis (especially on the soles of the feet);
➢ Incidence of cancers (skin—basal and squamous cell carcinoma - and osteosarcoma);
➢ Nail abnormalities (dysplastic or malformed);
➢ Skeletal abnormalities (forearm bones, absent or hypoplastic patella, osteopenia);
➢ Small size;
➢ Sparse hair (scalp, eyelashes and/or eyebrows).

### 2.1 Genetic basis of Rothmund-Thomson syndrome

Over 100 years after the first description of the disease, Kitao et al. (1999) identified *RECQL4* (located on chromosome 8q24.3) as the first gene associated with RTS, but already in this study it was evident that RTS presented locus heterogeneity. Further to this discovery, it was established that while *RECQL4* mutations are observed in ca.60% of RTS patients, a further 40% did not have variants identified in this gene, such that the disease was subdivided into RTS type 1 (no variants identified in *RECQL4*) and type 2 (biallelic variants in *RECQL4*) (Wang et al., 2003; Larizza et al., 2010; Wang and Plon, 2020). Over the years, it has become evident that there were also clinical differences between the disease subtypes, with RTS type 1 more likely to be associated with bilateral juvenile cataracts, while type 2 RTS is characterized by an increased risk for osteosarcoma and skeletal abnormalities (Wang et al., 2001; 2003; Mehollin-Ray et al., 2008).

Despite the revolution in the diagnosis of rare human genetic disorders afforded by next-generation sequencing techniques, the identification of new RTS genes remained stagnant for almost two decades, until the recent identification of three new genes associated with the disease: *ANAPC1* (located on chromosome 2q13)*, CRIPT* (located on chromosome 10q21.3) and *DNA2* (located on chromosome 2p21).

In 2019, Ajeawung et al., 2019 evaluated a cohort of ten individuals from seven families, three of them of Amish ancestry, with clinical findings compatible with the diagnosis of RTS type I (mainly poikiloderma, with other ectodermal findings, and juvenile cataracts). Pathogenic/likely pathogenic variants in *RECQL4* were ruled out previously by molecular studies. Exome-sequencing was performed in all probands and biallelic variants were identified in *ANAPC1*, followed by the identification of a single large region of homozygosity on chr2q13-q14.1 in the Amish individuals and low levels of *ANAPC1* expression levels in qRT-PCR experiments. A recurrent intronic variant (c.2705–198C>T) in *ANAPC1* was identified in all individuals, in a homozygous state in all Amish and in one non-Amish patient, and in compound heterozygosity with a loss-of-function (LoF) variant in the other ones. Three other individuals from two families presenting RTS, but without juvenile cataracts, and negative for variants in *RECQL4*, were also screened for *ANAPC1* variants, but the analysis failed to show causative variants in the gene, suggesting that it could be exclusively associated with RTS type I.

Although the number of individuals evaluated was small, the authors noticed a more complex phenotype in those individuals harboring the intronic variant *in trans* with a LoF allele, compared to the homozygotes for the intronic variant, with the former more frequently presenting growth compromise requiring growth hormone therapy, as well as a greater involvement and findings in teeth, nails, and endocrine, genital and skeletal systems (Ajeawung et al., 2019). An additional individual, a 14-month-old boy presenting poikiloderma and other ectodermal findings, as well as developmental delay, was reported in 2021 harboring the same intronic variant reported previously by Ajeawung et al. (2019) *in trans* with a 1.7 Mb microdeletion in 2q13 encompassing, among ten genes in this chromosomal region, *ANAPC1* (Zirn et al., 2021).

Interestingly, *ANAPC1* mutations account for only 10% of RTS patients, such that ca.30% of patients remained undiagnosed (Wang and Plon, 2020). These results showed that there must be other gene(s) responsible for the clinical features fulfilling the criteria for RTS. Fortunately, four years later, two new genes were described for the syndrome.

Averdunk et al. (2023) evaluated two individuals presenting short stature, feeding difficulties, poikiloderma, sparse hair/eyebrows and eyelashes, developmental delay, facial dysmorphisms, skeletal anomalies (osteopenia, metaphyseal striations, short terminal phalanges) and one of them developed cataracts at the age of 8 years. As they fulfill the clinical criteria established for this disorder (Wang et al., 2001), the patients were diagnosed as RTS. No variants in *RECQL4* and *ANAPC1* were identified, but both patients harbored homozygous variants in *CRIPT*. The authors also updated the clinical findings of four previously described individuals harboring variants in *CRIPT* (Shaheen et al., 2014; Leduc et al., 2016; Akalin et al., 2023). Thus, in the six individuals evaluated, all presented with neurologic involvement with developmental delay, particularly a severe speech compromise, and high prevalence of seizures; growth compromise with short stature and microcephaly; ectodermal findings, such as poikiloderma or hypopigmented/dyspigmented skin patches, sparse/absent hair, eyebrows and eyelashes, and less often, dysplastic nails; ophthalmologic abnormalities, mainly retinal pigmentary changes and one individual with cataracts; skeletal anomalies, mainly osteopenia, metaphyseal striations, short phalanges, scoliosis; recurrent pulmonary infections (leading to a premature death in one of the individuals) and no history of cancer development. The authors concluded that biallelic variants in *CRIPT* were responsible for a phenotype similar to RTS with neurologic impairment.

Our group was responsible for the discovery of another gene related to RTS (Di Lazzaro Filho et al., 2023). By the analyses of an admixed cohort, a specific cluster of patients displayed clinical symptoms reminiscent of RTS, but with a distinct genetic basis. Six individuals originated from Brazil and a duo of Swiss/Portuguese siblings, exhibited a phenotype marked by widespread poikiloderma, severe growth failure (with growth hormone—GH—deficiency or combined pituitary hormone deficiency), microcephaly and congenital cataracts. Additionally, these individuals exhibited clinical characteristics consistent with RTS, including sparse hair, eyebrows and eyelashes, dystrophic nails, craniofacial dysmorphisms and skeletal anomalies (osteopenia, metaphyseal flaring and short metacarpals/phalanges). Through whole genome sequencing (WGS), we identified compound heterozygosity for an intronic splicing variant *in trans* with LoF variants in *DNA2*, resulting in diminished DNA2 protein levels. Remarkably, this intronic variant was shared among all the patients and was also detected in the Portuguese father of the European siblings, suggesting a possible founder effect.

Thus, four different genes have been reported to be responsible for a phenotype typical or reminiscent of RTS. The number of individuals harboring variants in these novel genes is small, which precludes a clear genotype-phenotype correlation at present. However, a comparison between clinical findings observed for RTS patients harboring mutations in each of these genes highlights commonalities and gene-specific discrepancies in clinical presentation (Table 1). As expected, poikiloderma is present in all patients, although *DNA2-*patients have a more generalized distribution compared to other gene groups. Perhaps more surprising is the finding that both sparse hair/eyebrows/eyelashes and short stature are highly prevalent in all RTS gene groups. Skeletal findings such as short metacarpals/phalanges are also very prevalent, but *RECQL4* patients are much more prone to radial ray defects. Moreover, cataracts were almost exclusively seen in individuals harboring variants in *ANAPC1* and *DNA2*, in the former as the classical, juvenile form described in the first reports of RTS, and in the latter, as a congenital form, frequently associated with other ocular anomalies. Interestingly, the *ANAPC1* and *DNA2* groups share a high prevalence of growth-hormone deficiency. Neurological involvement, as either developmental delay/intellectual disability, or the presence of seizures, were most common in the individuals harboring *CRIPT* biallelic variants, while microcephaly is most frequent in both *CRIPT* and *DNA2* patients. The presence of osteosarcoma was seen only in individuals harboring biallelic variants in *RECQL4*, but given that the individuals in each group are still young, a long-term follow-up is required to determine whether the increased risk for cancer development is restricted to the *RECQL4* group.

**TABLE 1:** Clinical findings in individuals presenting RTS spectrum harboring variants in *RECQL4* (data from our group, not published), *ANAPC1*, *CRIPT* and *DNA2*. Symptoms marked in green are observed in >50% of patients harboring mutations in any of the four genes, while findings marked in orange are gene-specific phenotypes seen in >50% of patients with mutations in only one of the four genes.

	*RECQL4*	*ANAPC1*	*CRIPT*	*DNA2*
N^o^ of individuals/families	43/39	11/8	6/6	8/7
Gender	23F/20M	5F/6M	2F/4M	3F/5M
Age range	16 m—39 yo	NA	70 days—11 yo	2–18 yo
**Anthropometric measurements**				
Short stature (prenatal)	34/43	7/11 (0/1)	6/6	7/7 (6/7)
Z-score range	NR	NR	−2.89 to −5,3	−5,64 to −8,77
Microcephaly	NR	NR	5/6	6/7
Z-score range				−1.75 to—5.49
**Ophthalmologic findings**				
Cataracts	0/26	10/10 (juvenile)	1/6	7/7 (6/7 congenital)
Retinal/optic nerve anomalies	0/23	-	4/6	1/7
Microphthalmia	0/23	1/11	NR	4/7
Corneal opacity	0/23	-	NR	5/7
**Ectodermal findings**				
Poikiloderma (generalized)	41/43	11/11	4/4	7/7 (7)
Sparse hair/eyebrows and/or eyelashes	29/43	10/11	5/5	7/7
Dystrophic/small nails	4/7	5/11	2/3	6/7
**Skeletal Findings**				
Osteopenia/metaphyseal striations or irregular, wide metaphysis	7/23	2/5	6/6	2/3
Short metacarpals/phalanges	Short hands and/or feet (7/12)	2/5	5/6	4/4
Radial-ray defects	14/40	-	1/6 (proximally placed thumb)	0/7
Axial abnormalities	1/14	NR	6/6—scoliosis	2/2 mild platyspondyly
			2/6 platyspondyly	
**Endocrine anomalies**				
Growth hormone deficiency (GH therapy)	NR	5/10 (6)	-	6/6 (5)
Hypothyroidism	NR	2/10	-	5/6
**Genital anomalies**				
Cryptorchidism	NR	5/5	1/4	3/4M
Micropenis	0/1	1/5	-	4/4M
**Neurological findings**				
Developmental delay/intellectual disability	4/27	2/10	6/6	2/7
Seizures	NR	NR	4/6	0/7
**Other**				
Recurrent pulmonary infections	NR	3/10 (otitis media)	6/6 (pulmonary)	0/7
Cancer (type)	8/43 (6 osteosarcoma +2 skin cancer)	0/10	0/6	0/7
Death in childhood (cause)	1/43 (NR)	0/10	1/6 (pneumonia)	0/7
References	Cabral et al. (2008), Siitonen et al. (2009), Piard et al. (2015), Suter et al. (2016), Wang et al. (2018), Colombo et al. (2018), Zhang et al. (2021)	Ajeawung et al. (2019), Zirn et al. (2021)	Shaheen et al. (2014), Leduc et al. (2016), Akalin et al. (2023), Averdunk et al. (2023)	Di Lazzaro Filho et al. (2023)

### 2.2 Rothmund-Thomson syndrome variants and their impacts on gene function

**FIGURE 1:**
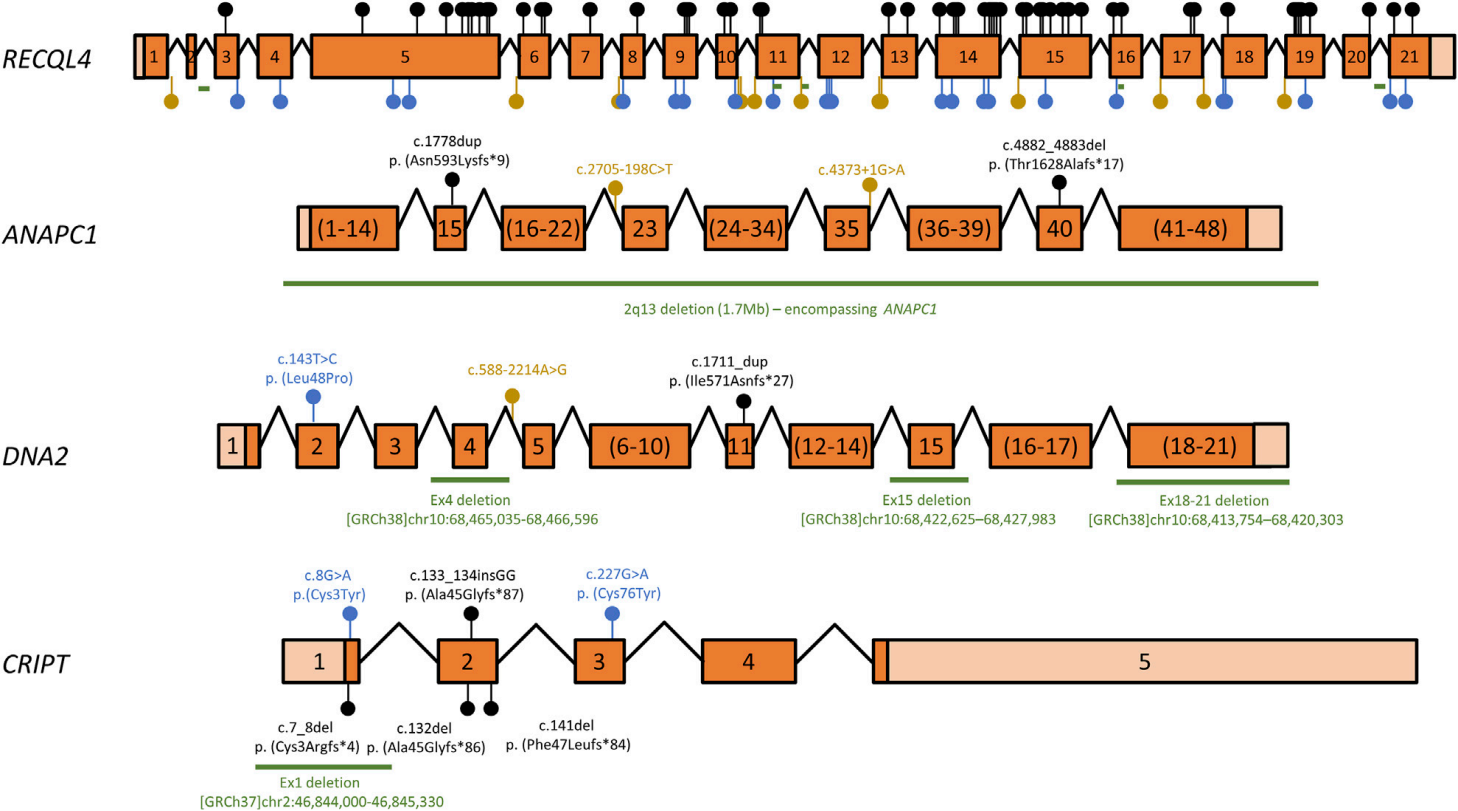
Overview of *RECQL4*, *ANAPC1*, *DNA2*, and *CRIPT* variants associated with RTS. Schematic representation of RTS gene cDNAs mapping known disease-associated variants. Exons are numbered, coding regions are dark orange and untranslated regions are shown in light orange. Exon sizes are shown to scale for each gene, but the scale ratio varies for different cDNAs. Exons numbered in brackets have been pooled for clarity and are not to scale. Introns are depicted as angled lines and are also not to scale. Variants that lead to premature termination codons (PTCs), i.e., nonsense mutations and insertion/deletion variants that lead to frameshifts, are shown as black lollipops. Missense mutations or in-frame insertions/deletions are shown as blue lollipops, while splice-site mutations or intronic variants are depicted as yellow lollipops. Deletions >20 nt are shown as green lines. A complete list of all variants is shown in Supplementary Table S01. cDNA structure and variant numbering are based on transcripts ENST00000617875 (*RECQL4),* ENST00000341068 (*ANAPC1*), ENST00000358410 (*DNA2)* and ENST00000238892 (*CRIPT*).

Over the years, several mutant alleles have been cataloged for RTS-related genes. Of these, a large proportion (87/114, or 76%) consist of variants that are either large deletions or are predicted to lead to premature termination codons (PTCs) or splicing defects (Figure 1 and Supplementary Table S1). As these aberrant transcripts are often degraded by quality control mechanisms such as nonsense-mediated mRNA decay (Lindeboom et al., 2019), most are predicted to lead to reduced mRNA and therefore low overall protein levels, such that the location of these mutations in relation to protein domain architecture or catalytic activities must be interpreted with caution. Even within the remaining missense or in-frame insertion/deletion mutations, which only result in localized changes to amino acid sequence, some variants may compromise overall protein stability, for instance by destabilizing protein folding, and their locations must therefore also be cautiously interpreted. However, some variants are particularly informative and will be briefly discussed. For instance, mRNAs containing PTCs within the last exon (exon 21 in the case of *RECQL4*), are generally spared of nonsense-mediated decay (Lindeboom et al., 2019), suggesting that the two *RECQL4* variants c.3523C>T (p. Gln1175*) and c.3599_3600delCG (p.Thr1200Argfs*26), which affect exon 21, may produce essentially full-length proteins, at essentially normal levels. While speculative, this would indicate that the very C-terminal portion of the 1208 amino acid-long RECQL4 protein may have important functions. Another interesting observation is that both missense mutations in *CRIPT* are cysteine-to-tyrosine mutations. Cysteines often participate in coordination of metal ions, such as in zinc finger domains. Indeed, upon close inspection of a recently published cryo-electron microscopy structure containing CRIPT among many other proteins (PDB: 7DVQ), the affected residues (cysteine 3 and cysteine 76) are part of two different Zn^2+^-coordinating centers in CRIPT, one formed by Cys3, Cys6, Cys83 and Cys86 and the other formed by Cys58, Cys61, Cys73 and Cys76 (Bai et al., 2021).

### 2.3 Other rare syndromes related to Rothmund-Thomson syndrome genes

#### 2.3.1 *RECQL4*

Biallelic variants in *RECQL4* have been associated, besides RTS, with two other distinct phenotypes, RAPADILINO and Baller-Gerold syndromes. These disorders present variable expressivity and there is a significant clinical overlap between them, which precludes a clear distinction on several occasions.

RAPADILINO syndrome (OMIM # 266280) is a very rare autosomal recessive disorder, described in 1989 in a pair of siblings and three non-related Finnish individuals. The acronym RAPADILINO was proposed for its main clinical features: RA for RAdial ray anomalies, PA for PAtella hypoplasia/aplasia, DI for DIarrhea and DIslocated joints, LI for LIttle size and LImb malformations and NO for long, slender NOse and NOrmal intelligence (Kääriäinen et al., 1989). The main differential diagnosis at the time of publication included the classical syndromes presenting with radial ray anomalies, particularly thrombocytopenia-absent radii syndrome (TAR), Holt-Oram syndrome, VATER association, and Fanconi anemia. Later, based on a clinical overlap between RAPADILINO syndrome and RTS, the Finnish individuals were screened for variants in *RECQL4* and the identification of biallelic variants in these individuals showed that RAPADILINO and RTS were allelic conditions (Siitonen et al., 2003).

This syndrome was reported almost exclusively in the Finnish population, in which a founder variant in *RECQL4*:c.1390+2delT:p.Ala420_463del was identified in the majority of these individuals, mainly in a homozygous state (Siitonen et al., 2003; 2009). The allele frequency of the variant c.1390+2delT in Finnish individuals is 108/24962 (0.004327), contrasting with a much lower prevalence of this variant in non-Finnish Europeans (data from GnomAD—https://gnomad.broadinstitute.org/). In 17 probands with biallelic variants in *RECQL4* and a diagnosis of RAPADILINO syndrome reported in the literature, the variant c.1390+2delT was present in 20 out of 34 alleles (59%), in accordance with a high prevalence of this disorder in the Finnish population and probably contributing to a more homogeneous phenotype (Supplementary Table S2).

The cardinal features reported initially are seen in a high frequency in individuals with proven biallelic variants in *RECQL4*, particularly growth compromise, preaxial upper limb defects and patellar anomalies, diarrhea and/or feeding problems, normal neurodevelopment and cognition and palatal anomalies. The absence of poikiloderma is particularly noteworthy in these individuals and has been considered the main clinical discriminator between RAPADILINO syndrome and RTS (Siitonen et al., 2003; 2009). Of note, six out of 15 Finnish individuals (40%) in a long-term follow-up developed osteosarcoma (2 individuals) or lymphoma (4 individuals) (Siitonen et al., 2009).

Baller-Gerold syndrome (BGS—OMIM # 218600) is another very rare autosomal recessive disorder, described in the 50′s by Baller (1950), and Gerold (1959), in individuals presenting craniosynostosis and radial ray abnormalities. The eponym Baller-Gerold syndrome was coined by Cohen in the 70′s (Cohen, 1975). Further reports of BGS included a series of additional clinical findings, including dysmorphic facial features (downslanting palpebral fissures, epicanthic folds, prominent nasal bridge, microstomia, micrognathia, abnormal ears); anal anomalies; cardiovascular, genitourinary, other skeletal (vertebral, lower limbs) and central nervous system anomalies and intellectual disability. The clear overlap of BGS with several well-known syndromes lead to a rediagnosis on several occasions in the literature, particularly as Fanconi anemia and Roberts syndrome, but also as Saethre-Chotzen syndrome, as well as the maternal use of valproate during pregnancy in individuals presenting a phenocopy of BGS (van Maldergem et al., 2006). These facts raised the question if BGS was indeed a distinct entity and some researchers suggested that the diagnosis of BGS should be restricted to the presence of the original described cardinal features (Cohen and Toriello, 1996).

The first report of biallelic variants in *RECQL4* in individuals with BGS was made by van Maldergem et al. (2006) in two families, one of them with four affected siblings. Thus far, 11 probands have been reported with BGS with proven variants in *RECQL4* (Supplementary Table S2). The main clinical findings were radial ray defects followed by craniosynostosis, particularly of the coronal or coronal and lambdoid sutures. Other less common features included growth compromise of prenatal onset, poikiloderma, sparse hair, eyebrows and eyelashes, anal anomalies, foot deformities/anomalies, palatal anomalies, and joint dislocations. One individual developed an extranodal NK/T-cell lymphoma at the age of 2 years and 5 months. Some of the reported individuals died prematurely or were the product of termination of pregnancy, precluding a thorough evaluation of clinical findings, some of which could only be evident during clinical evolution, such as poikiloderma (van Maldergem et al., 2006; Debeljak et al., 2009; Siitonen et al., 2009; Cao et al., 2015; Kaneko et al., 2017).

A precise genotype-phenotype correlation could not be established between RTS, RAPADILINO and BGS. More than 100 variants have been reported in *RECQL4* in individuals presenting mainly RTS, and less frequently, RAPADILINO and Baller-Gerold syndromes. Some of these variants are common to more than one phenotype (Schmit and Bielinsky, 2021; Xu et al., 2021). Thus, the lack of a clear genotype-phenotype correlation between the disorders related to *RECQL4*, in addition to narrow clinical discriminators between them, lead some authors to consider them within a phenotypic spectrum (Debeljak et al., 2009; Sznajer et al., 2009; Piard et al., 2015). Nonetheless, the distinct eponyms/acronym for these disorders are still being employed in recent broad revisions (Unger et al., 2023).

#### 2.3.2 *DNA2* and *CRIPT*

The first reports of individuals harboring biallelic variants in *DNA2* and *CRIPT* were identified in cohorts of individuals with a clinical diagnosis of primordial dwarfism.

Primordial dwarfism (PD) is a group of rare genetic conditions characterized by severe growth restrictions, both prenatally and postnatally, resulting in extremely small stature for age and often accompanied by microcephaly. PD includes, among other syndromes, Seckel syndrome, microcephalic osteodysplastic primordial dwarfism types 1 and 2 (Shaheen et al., 2014; Leduc et al., 2016; Tarnauskaitė et al., 2019; Akalin et al., 2023).

In the first reports of individuals with biallelic variants in *CRIPT*, growth compromise was a universal clinical finding, but a typical poikiloderma was not mentioned, although mottled hypopigmented skin or areas of hypopigmentation/hyperpigmentation that started in face and spread to upper and lower limbs were observed (Shaheen et al., 2014; Leduc et al., 2016). It was only recently that Averdunk et al. (2023) reported two individuals presenting several ectodermal findings including poikiloderma, sparse hair, along with skeletal findings, resembling RTS. The clinical findings of these individuals are depicted in Table 1.

In the first two reports of individuals harboring biallelic variants in *DNA2* **,** the clinical diagnosis included Seckel syndrome (Shaheen et al., 2014) and microcephalic primordial dwarfism (MPD)**/**Seckel syndrome 8 (SCKL8, OMIM # 615807) (Tarnauskaitė et al., 2019). In these reports, no reference to poikiloderma or skeletal abnormalities were included. Although two out of four individuals reported by Tarnauskaitė et al. (2019) presented sparse hair, no additional findings compatible with the diagnosis of RTS were reported. Therefore, these individuals were not included in Table 1 along with the individuals reported by our group with characteristics reminiscent of RTS (Di Lazzaro Filho et al., 2023). The mechanisms explaining the phenotypic differences between *DNA2*-related RTS and *DNA2*-related MPD remain to be determined.

In addition, heterozygous missense variants in *DNA2* were reported in individuals presenting a slowly progressive neuromuscular disorder characterized by muscle weakness, external ophthalmoplegia, exercise intolerance, and mitochondrial DNA (mtDNA) deletions on muscle biopsies (Progressive external ophthalmoplegia with mitochondrial DNA deletions—OMIM #615156) (Ronchi et al., 2013; Ronchi et al., 2019). This condition manifests typically in early adulthood. Patients present with exercise intolerance, myalgia, and cramping, which often lead to progressive myopathy. Muscle weakness commonly starts in the proximal limb muscles. Muscle biopsies usually reveal ragged-red fibers, indicative of mitochondrial dysfunction, and increased levels of deleted mtDNA, highlighting the role of DNA2 in maintaining mitochondrial genome stability, particularly in muscle cells. Abnormal creatine kinase levels and irregular electromyographic are also findings discovered during further evaluation (Ronchi et al., 2013; Ronchi et al., 2019). It is noteworthy that myopathy or other muscular alterations have not been described in either *DNA2*-related RTS or Seckel Syndrome 8.

## 3 Molecular activities of Rothmund-Thomson syndrome gene products

### 3.1 *RECQL4*

*RECQL4* encodes a member of the RECQ helicase family characterized by the presence of an ATP-dependent DNA helicase domain, with a 3′→5′polarity, first identified in *Escherichia coli* RecQ (Umezu et al., 1990). These proteins act in different cellular processes such as replication, recombination, repair, telomere maintenance, translation, RNA processing, mitochondrial DNA maintenance and chromosomal segregation, and are therefore important for the maintenance of genome stability (Chu and Hickson, 2009; Croteau et al., 2014; Wang and Plon, 2020; Balajee, 2021).

Uniquely among human RECQ family members, *RECQL4* is an essential gene. Interestingly, this essential function is not associated with helicase activity, but has instead been mapped to an N-terminal Sld2-like domain which is crucial for the initiation of DNA replication (Sangrithi et al., 2005; Matsuno et al., 2006; Im et al., 2009; Abe et al., 2011; Croteau et al., 2012a; Collart et al., 2013; Smeets et al., 2014; Castillo-Tandazo et al., 2021). During replication origin firing, RECQL4 is thought to promote the recruitment of the GINS complex and of the leading strand polymerase Polε to the prereplication-complex (pre-RC), in concert with TopBP1, and may also play roles in the subsequent recruitment of AND-1, Mcm10 and the DNA pol α-primase complex (Sangrithi et al., 2005; Matsuno et al., 2006; Im et al., 2009; Xu et al., 2009; Shin et al., 2019). Shin et al. (2019) demonstrated that direct tethering of RECQL4 to the pre-RC generated replication stress and the accumulation of single-stranded DNA (ssDNA) due to increased DNA synthesis in early S phase, suggesting that RECQL4 also regulates the temporal control of late replication origin firing and protects against replication stress.

In addition, *RECQL4* is thought to function in the maintenance of telomeres (Ghosh et al., 2009; 2012; Croteau et al., 2012a; Ferrarelli et al., 2013), which is evidenced by telomere abnormalities in *RECQL4*-mutated patient cells, such as telomere fragility and segmental exchanges between telomeric sister chromatids (Ghosh et al., 2012), as well as an increase in replication stress markers, such as 53BP1 bodies, localizing to telomeric structures in these cells (Ghosh et al., 2012). During S phase, the localization of RECQL4 to telomeres is increased, and it has been shown to interact directly with both telomeric DNA and telosome proteins (Ghosh et al., 2012). While the molecular roles of RECQL4 at telomeres are insufficiently understood, there is evidence that it can resolve telomeric D-loops, in particular those containing lesions such as 8-oxodG or thymine glycol, with the help of TRF1, TRF2 and POT1 (Ghosh et al., 2009; Ghosh et al., 2012; Ferrarelli et al., 2013).

RECQL4 has also been implicated in the replication and protection of the mitochondrial genome (Croteau et al., 2012b; Chi et al., 2012; De et al., 2012; Gupta et al., 2014). In this context, RECQL4 is thought to associate with p53 and serve as an accessory factor that stabilizes the interaction between subunits of the mitochondrial DNA polymerase Polγ, thus promoting the processivity of the PolγA/B2 holoenzyme (Gupta et al., 2014). Loss of both RECQL4 and p53 was shown to cause a decrease in the replication fidelity of mitochondrial genetic material, resulting in polymorphisms and somatic mutations (Gupta et al., 2014), although how this can be reconciled with the lack of clear mitochondria-related clinical symptoms in RTS patients is unclear.

The RECQL4 helicase domain has been shown to participate in the resection of DNA double-stranded breaks (DSB) during the repair of these lesions through homologous recombination (HR) (Xu and Liu, 2009; Croteau et al., 2012a; Lu et al., 2016), via a physical interaction of RECQL4 with the MRN (MRE11-RAD50-NBS1) complex and CtIP, responsible for initiating the resection of DSBs (Lu et al., 2016). RECQL4 also interacts with XPA, an important protein in nucleotide excision repair (NER), and this interaction is enhanced after UV-irradiation (Fan and Luo, 2008). However, since UV hypersensitivity is not a characteristic of RTS, the role of RECQL4 in NER should be secondary and needs further study (Fan and Luo, 2008). RECQL4 also interacts with proteins involved in base excision repair (BER), such as APE1 and FEN1, colocalizing after treatment with H_2_O_2_ and stimulating their apurinic endonuclease and incision activity, respectively (Schurman et al., 2009). Furthermore, RTS patients accumulate more XRCC1 foci and have difficulty responding normally to oxidative stress, indicating that RECQL4-deficient cells show an increased amount of oxidative damage (Schurman et al., 2009). Woo et al. (2006) also showed that RECQL4 accumulates in nucleoli after treatment with H_2_O_2_ and streptonigrin, interacting with PARP-1, a protein important in DNA damage signaling (Chiarugi, 2002; Huang et al., 2022). This nucleolar localization of RECQL4 is PARP-1 dependent, as it is abolished after the use of PARP-1 inhibitor. Interestingly, the C-terminal portion of RECQL4 is targeted by PARP-1 (Woo et al., 2006).

Therefore, despite being extensively studied, the precise biochemical role of RECQL4 in DNA replication, DNA repair and how defects in its activity lead to the clinical presentations of RTS still lacks characterization.

### 3.2 *ANAPC1*

*ANAPC1* encodes the largest subunit of the anaphase-promoting complex/cyclosome (APC/C), which is an E3 ubiquitin ligase that targets proteins for degradation by the proteasome. Its main functions are in the control of the cell cycle, both during mitosis and in the G1/S transition (Melloy, 2020).

At the end of mitosis and during G1, the APC/C is bound to the co-activator Cdh1 and high APC/C^Cdh1^ complex activity is required to maintain cells in G1 and allow the licensing of new replication origins in preparation for the next S phase (Harper et al., 2007; Sigl et al., 2009; Eguren et al., 2011; Cappell et al., 2016; Schrock et al., 2020). APC/C^Cdh1^ inactivation is a crucial step during the transition between the G1 and S phases and is considered the “*point of no return*” for the onset of S phase (Cappell et al., 2016). Furthermore, the absence of Cdh1 leads to incomplete DNA replication (Greil et al., 2016), probably due to dysregulation in dNTP levels and a consequent decrease in replication fork speed (Garzón et al., 2017).

APC/C^Cdh1^ has also been shown to have non-canonical functions in response to DNA damage, and the loss of Cdh1 increases genome instability and sensitivity to DNA damaging agents (Engelbert et al., 2008; Lafranchi et al., 2014). In G2, APC/C^Cdh1^ prevents the initiation of mitosis after DNA damage via degradation of the mitotic cyclins A and B (Sudo et al., 2001; Peters, 2006; Bassermann et al., 2008). In G1, APC/C^Cdh1^ interacts with CtIP, targeting it for degradation and restricting DNA end resection and HR efficiency, while in G2 and after DNA damage, this activity is regulated by the SKP2-SCF complex to promote HR under these conditions (Lafranchi et al., 2014; Li et al., 2020).

APC/C can also be activated by Cdc20, which occurs during mitosis and ensures the progression from metaphase to anaphase, by promoting the degradation of securin, Pds1, and cyclin B, which allows the separation of sister chromatids and segregation of chromosomes to opposite poles of the cell during anaphase (Peters, 2006; Harper et al., 2007). In this context, APC/C^Cdc20^ is essential for correct chromosome segregation and cell division, and the inhibition of this complex causes a delay in progression through mitosis, with cells arrested in metaphase (Zeng et al., 2010).

While the importance of the APC/C complex, and therefore of the *ANAPC1* gene, in cell cycle progression and regulation of target protein levels in different cell cycle stages is well-established, how the loss of these functions may contribute to the phenotype of RTS patients is still unknown.

### 3.3 *DNA2*

Like *RECQL4*, the *DNA2* gene is embryonic lethal, which has made it difficult to elucidate its functions. DNA2 has two catalytic activities contained in distinct active sites: an ATP-dependent helicase domain with 3′→5′polarity and an endonuclease domain (Budd and Campbell, 1995; Bae et al., 1998; Liao et al., 2008).

Due to these capabilities, DNA2 has been implicated in multiple DNA replication and repair mechanism. In budding yeast, DNA2 was shown to participate in the maturation of Okazaki fragments during lagging strand replication (Kang et al., 2010), although there is little evidence for a similar activity in humans (Duxin et al., 2012). During lagging strand replication, the yeast Pif1 helicase, together with the Polδ (delta) subunit, Pol32, unwind previously synthesized Okazaki fragments. If these fragments are short (<30 nt), they are readily cleaved by the canonical flap endonuclease FEN1 (Kang et al., 2010; Hudson and Rass, 2021). However, if longer flaps (>30 nt) are generated, these are covered by RPA, inhibiting the action of Fen1 and requiring DNA2 to incise the flap and remove a large portion of the ssDNA region, allowing Fen1 to process the resulting short flap (Bae and Seo, 2000; Bae et al., 2001; Rossi et al., 2018; Kahli et al., 2019). In the absence of DNA2, these RPA-covered flaps are thought to activate DNA damage signaling via Mec1 (ATR), which likely accounts for the lethality of DNA2 mutants (Budd et al., 2011; Rossi et al., 2018). Although DNA2 also contains a helicase domain, its functions are less clear, given that DNA2 often associates with other helicases such as WRN (RECQL2) and/or BLM (RECQL3) (Masuda-Sasa et al., 2006).

DNA2 is also important during replication fork reversal (Neelsen and Lopes, 2015; Hudson and Rass, 2021), which is a process by which DNA replication forks are remodeled to allow their restart after their progression has been blocked (Zellweger et al., 2015). DNA2 is recruited to stalled replication forks and is required for controlled nucleolytic degradation of reversed replication forks, in concert with WRN, which promotes replication restart and prevents the aberrant processing of replication intermediates by other pathways (Hu et al., 2012; Neelsen and Lopes, 2015; Thangavel et al., 2015; Hudson and Rass, 2021).

DNA2 also plays a role in the replication of repetitive regions of the genome, such as centromeres and telomeres, requiring both of its functions (helicase and endonuclease) for the resolution of structures formed in these regions, and thus facilitating replication fork progression (Choe et al., 2002; Lin et al., 2013; Li et al., 2018). At telomeres, DNA2 has been proposed to process G4 quadruplex structures formed in the G-rich strand (Masuda-Sasa et al., 2008; Lin et al., 2013), C-rich ssDNA (Markiewicz-Potoczny et al., 2018), and to promote *de novo* telomere addition by telomerase (Choe et al., 2002). At centromeres, it has been proposed that DNA2 is also involved in resolving G4 structures and stem loops formed due to the repetitive nature of the centromeric DNA, perhaps in a similar fashion to its proposed role in flap processing during Okazaki fragment maturation in concert with FEN1 (Tishkoff et al., 1997; Li et al., 2018).

Perhaps the most well-established role of DNA2, at least in human cells, is its involvement in the repair of DSBs via homologous recombination (Zhu et al., 2008; Di Lazzaro Filho et al., 2023). In this process, the initial resection of the DSB regions is performed by the MRN complex, which degrades only a few nucleotides (short resection) (Zhu et al., 2008), activated by the CtIP protein. Subsequently, DNA2 combines with WRN or BLM (RECQL3) to perform long-range resection, which can also be performed in a redundant fashion by the exonuclease EXO1 (Budd et al., 2005; Mimitou and Symington, 2009; Nimonkar et al., 2011; Ranjha et al., 2018).

As with RECQL4 and ANAPC1, it is unclear which of these multiple functions of DNA2 could be most relevant for RTS pathology.

### 3.4 *CRIPT*

*CRIPT* (cysteine-rich interactor of PDZ three) is a well-conserved protein, being present from plants to animals and is expressed in several tissues (Niethammer et al., 1998), with highest expression in the hippocampus, moderately in the striatum and cortex and in smaller amounts in the midbrain (Niethammer et al., 1998). CRIPT is known for binding to the third domain of PSD-95 (PDZ3) (Niethammer et al., 1998), which localizes in specific cell-cell binding sites at synapses in the mammalian nervous system and is involved in cellular localization of proteins and in establishing cell polarity (Ehlers et al., 1996). By identifying activities for sub-membranous protein complexes, PDZ domains serve as a signaling mechanism for a variety of pathways as receptor-mediated, transport channel-mediated and cytoskeleton-associated molecules, binding to specific C-terminal regions of other proteins (Ponting et al., 1997). Both CRIPT and PSD-95 co-localize in excitatory synapses and dendritic spines, interacting with the tubulin portion of the cytoskeleton (Niethammer et al., 1998), with CRIPT being the bridge between PSD-95 and the microtubules, given that PSD-95 lacks this ability (Passafaro et al., 1999).

CRIPT is also involved in mRNA processing, by stabilizing U12 small nuclear ribonucleoprotein (snRNP) within the minor spliceosome complex (Bai et al., 2021). U12-introns are rare regions in the genome, which require a splicing machinery different from the machinery used in the removal of introns of the U2-type (more abundant) (Burge et al., 1998). This machinery is called U12-dependent or minor spliceosome and is believed to be evolutionarily ancient, given that U12-introns are present from cnidarians to mammals and plants, being absent in *C. elegans* and yeast (Wu et al., 1996; Dietrich et al., 1997; Burge et al., 1998).

Cells from *CRIPT* patients exhibit high expression of senescence markers (p53/p16/p21), confirmed by the ß-galactosidase activity test. Even so, no significant increase was observed in cell sensitivity to various genotoxic agents, such as ionizing radiation, mitomycin C, hydroxyurea, etoposide, and potassium bromate (Averdunk et al., 2023), precluding the identification of a role for CRIPT in DNA repair.

How these varied functions of CRIPT relate to the clinical presentation of RTS patients is currently unknown.

## 4 Is there a shared mechanism underlying Rothmund-Thomson syndrome pathology?

As evidenced by the variety of functions of RTS-related genes described above, there is currently no clear understanding for how variants in these four genes lead to a similar clinical presentation. While a role for most RTS genes in DNA replication, DNA repair and/or cell cycle progression points to a shared defect in at least one of these processes, the precise nature of this putative shared pathway remains to be clarified. One possibility is that these proteins share a common role in DSB end resection. In support of this, RECQL4 was found to interact with DNA2 and other DNA end resection factors, such as Mre11 and CtIP (Lu et al., 2016), and the APC/C has also been shown to interact with CtIP in this context (Lafranchi et al., 2014). Interestingly, mutations in CtIP have been identified in patients diagnosed with Seckel syndrome or Jawad syndrome that actually present overlapping symptoms to Rothmund-Thomson syndrome (Qvist et al., 2011; Shaheen et al., 2014). Another area of clear overlap in known roles for these proteins is in regulating DNA replication, be that for origin firing regulation, replication fork progression or during replication of repetitive sequences such as centromeres or telomeres. Given that ANAPC1 is part of a complex that regulates protein degradation and CRIPT has been implicated in splicing, another possibility is that these proteins regulate the expression or stability of other RTS gene products. For instance, some evidence indicates that RECQL4 protein levels are regulated during the cell cycle, and that the APC/C may promote RECQL4 degradation by the proteasome during the transition from mitosis to G1 (Ajeawung et al., 2019). In agreement with some form of cross-regulation between APC/C and RECQL4, the APC/C has recently been shown to interact with and regulate RECQL4 activity during the G1/S transition (Xu et al., 2023). Similarly, a quick search of the U12DB database (Genome Bioinformatics Research Lab, 2023) identified a U12-type intron in *DNA2*, suggesting that CRIPT may promote adequate splicing of the *DNA2* transcript.

## 5 Conclusion

The recent identification of three new genes associated with RTS has renewed the interest in understanding the molecular and pathophysiological mechanisms that underlie this complex disorder. However, these genes have no obvious shared function, such that further studies are required to assist in the definition of common pathways regulated by them. As often observed in the past, human genetics may be providing molecular and cellular biologists with important clues that could be critical to elucidate the molecular functions of human genes. Crucially, these discoveries may inform the development of improved clinical guidelines for the care of RTS patients in the future.
