# Supplementary material for: Rothmund-Thomson syndrome, a disorder far from solved
Source: Front Aging. 2023 Nov 10;4:1296409. doi: 10.3389/fragi.2023.1296409 (PMC10676203; doi:10.3389/fragi.2023.1296409)
Supplement: Supplementary file 1 [file Table1.DOCX]

Supplementary Material

**Rothmund-Thomson syndrome, a disorder far from solved**

**Davi Jardim Martins, Ricardo Di Lazzaro Filho, Debora Romeo Bertola^*^, Nícolas Carlos Hoch^*^**

***Correspondence:** Co-corresponding Authors

[debora.bertola@usp.br](mailto:debora.bertola@usp.br) [nicolas@iq.usp.br](mailto:nicolas@iq.usp.br)

# Supplementary Tables

Supplementary Table 1: List of variants identified in *RECQL4, ANAPC1, CRIPT* and *DNA2* genes associated with Rothmund-Thomson syndrome.

| ***RECQL4*** | | |  |
| --- | --- | --- | --- |
| **DNA change** | **Protein change** | **Reference** |  |
|  |  |  |  |
| **single nucleotide missense/nonsense variants** | | |  |
| c.212A>G | p.(Glu71Gly) | Wang et al. (2003) |  |
| c.308C>T | p.(Pro103Leu) |  |  |
| c.691G>A | p.(Gly231Ser) | Gui et al. (2018) |  |
| c.759G>T | p.(Gln253His) | Cabral et al. (2008) |  |
| c.792G>A | p.(Trp264*) | Powis et al. (2018) |  |
| c.910C>T | p.(Gln304*) | Wang et al. (2018) |  |
| c.1000G.T | p.(Glu334*) | Al-Shamsi et al. (2016) |  |
| c.1078C>T | p.(Gln360*) | Suter et al. (2016) |  |
| c.1089C>G | p.(Tyr363*) | Ceyhan-Birsoy et al. (2019) |  |
| c.1149G>A | p.(Trp383*) | Reix et al. (2007) |  |
| c.1222C>T | p.(Gln408*) | Suter et al. (2016) |  |
| c.1236G>A | p.(Trp412*) | Piard et al. (2015) |  |
| c.1397C>T | p.(Pro466Leu) | Jin et al. (2008) |  |
| c.1531T>C | p.(Cys511Arg) | Piard et al. (2015) |  |
| c.1564C>T | p.(Arg522Cys) | Wang et al. (2003) |  |
| c.1678A>T | p.(Lys560*) | Dang et al. (2009) |  |
| c.1697T>C | p.(Leu566Pro) | Suter et al. (2016) |  |
| c.1772C>T | p.(Pro591Leu) | Jin et al. (2008) |  |
| c.1913T>C | p.(leu638Pro) | Sznajer et al. (2008) |  |
| c.2161C>T | p.(Arg721*) | Cao et al. (2017) |  |
| c.2221G>A | p.(Ala741Thr) | Cabral et al. (2008) |  |
| c.2263C>T | p.(Arg755Trp) | Grelet et al. (2019) |  |
| c.2269C>T | p.(Gln757*) | Kitao et al. (1999) |  |
| c.2272C>T | p.(Arg758*) | Colombo et al. (2014) |  |
| c.2395G>A | p.(Val799Met) | Wang et al. (2003) |  |
| c.2398C>T | p.(Gln800*) | Siitonen et al (2009) |  |
| c.2428C>T | p.(Gln810*) | Wang et al. (2003) |  |
| c.2461C>T | p.(Gln821*) | Siitonen et al (2009) |  |
| c.2476C>T | p.(Arg826*) | Wang et al. (2003) |  |
| c.2590C>T | p.(Gln864*) | Piard et al. (2015) |  |
| c.2662C>T | p.(Gln888*) | Jin et al. (2008) |  |
| c.2719C>T | p.(Gln907*) | Wang et al. (2018) |  |
| c.2780T>G | p.(Leu927Arg) | Cabral et al. (2008) |  |
| c.2802G>A | p.(Trp934*) | Piard et al. (2015) |  |
| c.3062G>A | p.(Arg1021Gln) | Wang et al. (2003) |  |
| c.3061C>T | p.(Agr1021Trp) | Kellermayer et al. (2005) |  |
| c.3313G>A | p.(Gly1105Ser) | Jin et al. (2008) |  |
| c.3509C>T | p.(Pro1170Leu) | Wang et al. (2003) |  |
| c.3523C>T | p.(Gln1175*) |  |  |
| c.3573C>G | p.(Ser1191Arg) | Suter et al. (2016) |  |
| **splice site variants** | | |  |
| c.1132-2A>G | -- | Yadav et al. (2019) |  |
| c.1391-1G>A | -- | Lindor et al. (2000) |  |
| c.1704G>A | -- | Wang et al. (2003) |  |
| c.1705-1G>A | -- | Sznajer et al. (2008) |  |
| c.1704+1G>A | -- | Simon et al. (2010) |  |
| c.1878+5G>A | -- | Wang et al. (2003) |  |
| c.2059-1G>C | -- | Beghini et al. (2003) |  |
| c.2059-1G>C | -- | Kitao et al. (1999) |  |
| c.2464-1G>C | -- | Wang et al. (2003) |  |
| c.2886-2A>T | -- | Jin et al. (2008) |  |
| c.3054A>G | -- | Colombo et al. (2018) |  |
| c.3236G>T | -- |  |  |
| **small deletion variants** | | |  |
| c.84+6_84+21del16 | -- | Siitonen et al (2009) |  |
| c.1048_1049delAG | p.(Arg350Glyfs*21) | Wang et al. (2003) |  |
| c.1343_1347delCCACC | p.(Pro448Argfs*18) | Piard et al. (2015) |  |
| c.1568delG | p.(Ser523Thrfs*35) | Cabral et al. (2008) |  |
| c.1573delT | p.(Cys525Alafs*33) | Lindor et al. (2000) |  |
| c.1650_1656delGGCCTGC | p.(Ala551Tyrfs*5) | Kitao et al. (1999) |  |
| c.1718delA | p.Gln573Argfs*5) | Wang et al. (2003) |  |
| c.1724_1725delAC | p.(His575Argfs*7) | Gui et al. (2018) |  |
| c.1919_1941_TCACAG | p.(Leu640_Ala642delinsPro) | Simon et al. (2010) |  |
| c.2085delA | p.(Lys695Asnfs*148) | Suter et al. (2016) |  |
| c.2412_2420delGGCCGGGCG | p.(Ala805_Arg807del) | Colombo et al. (2018) |  |
| c.2492_2493delAT | p.(His831Argfs*52) | Kitao et al. (1999) |  |
| c.2547_2548delGT | p.(Phe850Profs*33) | Wang et al. (2003) |  |
| c.2552delC | p.(Pro851Leufs*97) |  |  |
| c.2767_2768delTT | p.(Leu923Alafs * 53) | Fradin et al. (2013) |  |
| c.3008_3009delTG | p.(Val1003Alafs*29) | Suter et al. (2016) |  |
| c.3021_3022delCT | p.(Cys1008Profs*24) | Colombo et al. (2018) |  |
| c.3072_3073delAG | p.(Val1026Alafs*6) | Wang et al. (2003) |  |
| c.3270delG | p.(Glu1090Aspfs*60) | Siitonen et al (2009) |  |
| c.3277delG | p.(Asp1093Metfs*57) | Wang et al. (2003) |  |
| c.3501_3502delCG | p.(Ile1167Metfs*59) |  |  |
| c.3599_3600delCG | p.(Thr1200Argfs*26) | Siitonen et al (2009) |  |
| **small insertion variants** | | |  |
| c.156_160dupGGGCC | p.(Gln54Argfs*31) | Wang et al. (2018) |  |
| c.558_564dupAGATCCT | p.(Gly189Argfs*11) | Suter et al. (2016) |  |
| c.1015dupC | p.(Leu339Profs*11) | Jin et al. (2008) |  |
| c.1453dupC | p.(Gln485Profs*55) | Bhoyrul et al. (2017) |  |
| c.1930_1935dupGCCACA | p.(Ala644Thr645dup) | Suter et al. (2016) |  |
| c.2211dupC | p.(Lys738Glnfs*71) | Wang et al. (2003) |  |
| c.2277dupC | p.(Phe760Leufs*49) | Suter et al. (2016) |  |
| c.2415_2419dupCGGGC | p.(Arg807Profs*38) | Grelet et al. (2019) |  |
| c.2415_2419insCGGGG | p.(Arg807Profs*38) | Siitonen et al (2009) |  |
| c.2421dupT | p.Asp808* | Gui et al. (2018) |  |
| c.2569_2574dupTGCACC | p.(Cys857_thr858dup) | Suter et al. (2016) |  |
| c.2638dupC | p.Gln880Profs*4) | Jin et al. (2008) |  |
| c.3284_3287dupAGCG | p.(Ser1097Alafs*14) | Cao et al. (2017) |  |
| c.3330dupA | p.(Glu1111Argfs*116) | Suter et al. (2016) |  |
| **small indel variants** | | |  |
| c.978_979delTCinsG | p.(Ser326Argfs*33) | Yadav et al. (2019) |  |
| c.1568_1573delGCCCCTinsCCCC | p.(Ser523Thrfs*35) | Suter et al. (2016) |  |
| c.3283delGinsCC | p.(Glu1095Profs*15) | Zhang et al. (2016) |  |
| **larger deletion variants** | | |  |
| c.118+27del25 | -- | Jin et al. (2008) |  |
| c.1770_1807del38 | -- | Gui et al. (2018) |  |
| c.1878+32_1878+55del24 | -- | Wang et al. (2003) |  |
| c.2789_2812del24 | -- | Suter et al. (2016) |  |
| c.3502+44_3502+96del53 | -- | Cao et al. (2017) |  |
|  | | |  |
| ***ANAPC1*** | | |  |
| **DNA change** | **Protein change** | **Reference** |  |
|  |  |  |  |
| **splice site variants** | | |  |
| c.2705-198C>T | -- | Ajeawung et al. (2019) |  |
|  |  | Zirn et al. (2021) |  |
|  |  | Schwieger-Briel et al. (2021) |  |
| **small deletion variants** | | |  |
| c.4882_4883del | p.(THhr1628Alafs*17) | Ajeawung et al. (2019) |  |
| **small insertion variants** | | |  |
| c.1778dupA | p.(Asn593Lysfs*9) | Ajeawung et al. (2019) |  |
| **larger deletion variants** | | |  |
| 2q13 Del | -- | Zirn et al. (2021) |  |
| **intronic variants** | | |  |
| c.4373+1G>A | -- | Ajeawung et al. (2019) |  |
|  | | |  |
| ***CRIPT*** | | |  |
| **DNA change** | **Protein change** | **Reference** |  |
|  |  |  |  |
| **single nucleotide missense/nonsense variants** | | |  |
| c.8G>A | p.(Cys3Tyr) | Leduc et al. (2016) |  |
| c.227G>A | p.(Cys76Tyr) | Averdunk et al (2023) |  |
| **small deletion variants** | | |  |
| c.7_8del | p.(Cys3ArgfsTer4) | Akalin et al. (2023) |  |
| c.132del | p.(Ala45Glyfs*86) | Averdunk et al (2023) |  |
| c.141delT | p.(Phe47Leufs*84) | Shaheen et al. (2014) |  |
| **small insertion variants** | | |  |
| c.133_134insGG | p.(Ala45Glyfs*87) | Shaheen et al. (2014) |  |
| **larger deletion variants** | | |  |
| Ex1 Del | -- | Leduc et al. (2016) |  |
|  | | |  |
| ***DNA2*** | | |  |
| **DNA change** | **Protein change** | **Reference** |  |
|  |  |  |  |
| **single nucleotide missense/nonsense variants** | | |  |
| c.143T>C | p.(Leu48Pro) | Di Lazzaro Filho et al. (2023) |  |
| **splice site variants** | | |  |
| c.588-2214A>G | -- | Di Lazzaro Filho et al. (2023) |  |
| **small insertion variants** | | |  |
| c.1711_dup | p.(Ile571Asnfs*27) | Di Lazzaro Filho et al. (2023) |  |
| **larger deletion variants** | | |  |
| Ex4 deletion | -- | Di Lazzaro Filho et al. (2023) |  |
| Ex15 deletion | -- |  |  |
| Ex18 deletion | -- |  |  |

Supplementary Table 2: Variants identified in *RECQL4* in individuals with a clinical diagnosis of RAPADILINO and Baller-Gerold syndromes.

| **RAPADILINO syndrome**  **Mutations reported** | N^o^ of families  17 | Reference |
| --- | --- | --- |
| c.1390+2delT/  c.1390+2delT | 8 | Siitonen et al. (2003, 2009) |
| c.1390+2delT/  c.3271C > T:p.Glu1091* | 1 | Siitonen et al., 2003 |
| c.1390+2delT/  c.806G > A:p.Trp269* | 1 | Siitonen et al., 2003 |
| c.1390+2delT/  c.3214A >T:p.Arg1072* | 1 | Siitonen et al., 2003 |
| c.1390+2delT/  c.3599_3600delCG:p.Thr1200Argfs*26 | 1 | Siitonen et al., 2009 |
| c.1573delT: p.Cys525Alafs*33/  c.2091T>G:p.Phe697Leu | 1 | Siitonen et al., 2009 |
| c.1910T>C: p.Phe637Ser/  c.2476C>T:p.Arg826* | 1 | Siitonen et al., 2009 |
| c.1885del:p.Arg629Serfs*60/ c.2269C>T:p.Gln757* | 1 | Siitonen et al., 2009 |
| c.2059-1G>A/  c.3072delA:p.Val1026Cysfs*18 | 1 | Siitonen et al., 2009 |
| c.1397C>T, p.Pro466Leu/  c.1887del:p.Glu630Alafs*59 | 1 | Siitonen et al., 2009 |
| **BGS**  **Mutations reported** | N^o^ of families  7 |  |
| c.3061C>T (p.Arg1021Trp)/  c.1573del | 1 | Van Maldergem et al., 2006 |
| c.3056-2A>C/  c.3056-2A>C | 1 | Van Maldergem et al., 2006 |
| c.2335_2356del/  c.2335_2356del | 1 | Siitonen et al., 2009 |
| c.496C>T:p.Gln166*/  c.3151A>G:p.Ile1051Val | 1 | Siitonen et al., 2009 |
| c.2492_2493del; p. His831Argfs*52/  c.2506_2518del:p.Thr836Trpfs*3) | 1 | Debeljak et al., 2009 |
| c.2059-1G>C/  c.2141_2142del | 1 | Cao et al. 2015 |
| g.145737562_145739175delinsC/  g.145737562_145739175delinsC | 1 | Kaneko et al., 2017 |

# References

Ajeawung, N.F., Nguyen, T.T.M., LU, L., Kucharski, T.J., Rousseau, J., Molidperee, S., et al. (2019). Mutations in ANAPC1, Encoding a Scaffold Subunit of the Anaphase-Promoting Complex, Cause Rothmund-Thomson Syndrome Type 1. Am J Hum Genet. 105(3), 625-630. doi: 10.1016/j.ajhg.2019.06.011

Akalin, A., Şimşek-Kiper, P.Ö., Taşkiran, E.Z., Karaosmanoğlu, B., Utine, G.E., Boduroğlu, K. (2023). A novel biallelic CRIPT variant in a patient with short stature, microcephaly, and distinctive facial features. Am J Med Genet A. 191(4), 1119-1127. doi: 10.1002/ajmg.a.63120

Al-Shamsi, A., Hertecant, J.L., Souid, A.K., Al-Jasmi, F.A. (2016). Whole exome sequencing diagnosis of inborn errors of metabolism and other disorders in United Arab Emirates. Orphanet J Rare Dis. 11(1), 94. doi: 10.1186/s13023-016-0474-3

Averdunk, L., Huetzen, M.A., Moreno-Andrés, D., Kalb, R., Mckee, S., Hsieh, T.C., et al. (2023). Biallelic variants in CRIPT cause a Rothmund-Thomson-like syndrome with increased cellular senescence. Genet Med. 25(7), 100836. doi: 10.1016/j.gim.2023.100836

Beghini, A., Castorina, P., Roversi, G., Modiano, P., Larizza, L. (2003). RNA processing defects of the helicase gene RECQL4 in a compound heterozygous Rothmund-Thomson patient. Am J Med Genet A. 120A(3), 395-399. doi: 10.1002/ajmg.a.20154

Bhoyrul, B., Lindsay, H., Robinson, R., Stahlschmidt, J., Palmer, T., Edward, S., et al. (2017). Pili annulati in a case of Rothmund-Thomson syndrome with a novel frameshift mutation in RECQL4. J Eur Acad Dermatol Venereol. 32(6), e221-e223. doi: 10.1111/jdv.14742

Cabral, R.E., Queille, S., Bodemer, C., De Prost, Y., Neto, J.B., Sarasin, A., et al. (2008). Identification of new RECQL4 mutations in Caucasian Rothmund-Thomson patients and analysis of sensitivity to a wide range of genotoxic agents. Mutat Res. 643(1-2), 41-47. doi: 10.1016/j.mrfmmm.2008.06.002

Cao, D.H., Mu, K., Liu, D.N., Sun, J.L., Bai, X.Z., Zhang, N., et al. (2015). Identification of novel compound heterozygous RECQL4 mutations and prenatal diagnosis of Baller-Gerold syndrome: a case report. Genet Mol Res. 14(2), 4757-4566. doi: 10.4238/2015.May.11.8

Ceyhan-Birsoy, O., Murry, J.B., Machini, K., Lebo, M.S., Yu, T.W., Fayer, S., et al. (2019). Interpretation of Genomic Sequencing Results in Healthy and Ill Newborns: Results from the BabySeq Project. Am J Hum Genet. 104(1), 76-93. doi: 10.1016/j.ajhg.2018.11.016

Colombo, E.A., Fontana, L., Roversi, G., Negri, G., Castiglia, D., Paradisi, M., et al. (2014). Novel physiological RECQL4 alternative transcript disclosed by molecular characterisation of Rothmund-Thomson Syndrome sibs with mild phenotype. Eur J Hum Genet. 22(11), 1298-1304. doi: 10.1038/ejhg.2014.18

Dang, L., Li, Y., Yu, S. (2009). Mutation analysis in RECQL4 gene in a patient with Rothmund-Thomson syndrome. Chinese Journal of Dermatology, 42(1), 28-30

Debeljak, M., Zver, A., Jazbec, J. (2009). A patient with Baller-Gerold syndrome and midline NK/T lymphoma. Am J Med Genet A. 149A(4), 755-759. doi: 10.1002/ajmg.a.32736

Di Lazzaro Filho, R., Yamamoto, G.L., Silva, T.J., Rocha, L.A., Linnenkamp, B.D.W., Castro, M.A.A., et al. (2023). Biallelic variants in DNA2 cause poikiloderma with congenital cataracts and severe growth failure reminiscent of Rothmund-Thomson syndrome. J Med Genet. jmg-2022-109119. doi: 10.1136/jmg-2022-109119. Epub ahead of print

Fradin, M., Merklen-Djafri, C., Perrigouard, C., Aral, B., Muller, J., Stoetzel, C., et al. (2013). Long-term follow-up and molecular characterization of a patient with a RECQL4 mutation spectrum disorder. Dermatology. 226(4), 353-357. doi: 10.1159/000351311

Grelet, M., Blanck, V., Sigaudy, S., Philip, N., Giuliano, F., Khachnaoui, K., et al. (2019). Outcomes of 4 years of molecular genetic diagnosis on a panel of genes involved in premature aging syndromes, including laminopathies and related disorders. Orphanet J Rare Dis. 14(1), 288. doi: 10.1186/s13023-019-1189-z

Gui, B., Song, Y.; Hu, X., Li, H., Qin, Z., Su, J., et al. (2018). Novel pathogenic RECQL4 variants in Chinese patients with Rothmund-Thomson syndrome. Gene. 654, 110-115. doi: 10.1016/j.gene.2018.02.047

Jin, W., Liu, H., Zhang, Y., Otta, S.K., Plon, S.E., Wang, L.L. (2008). Sensitivity of RECQL4-deficient fibroblasts from Rothmund-Thomson syndrome patients to genotoxic agents. Hum Genet. 123(6), 643-653. doi: 10.1007/s00439-008-0518-4

Kaneko, H., Izumi, R., Oda, H., Ohara, O., Sameshima, K., Ohnishi, H., et al. (2017). Nationwide survey of Baller‑Gerold syndrome in Japanese population. Mol Med Rep. 15(5), 3222-3224. doi: 10.3892/mmr.2017.6408

Kellermayer, R., Siitonen, H.A., Hadzsiev, K., Kestilä, M., Kosztolányi, G. (2005). A patient with Rothmund-Thomson syndrome and all features of RAPADILINO. Arch Dermatol. 141(5), 617-620. doi: 10.1001/archderm.141.5.617

Kitao, S., Shimamoto, A., Goto, M., Miller, R.W., Smithson, W.A., Lindor, N.M., et al. (1999). Mutations in RECQL4 cause a subset of cases of Rothmund-Thomson syndrome. Nat Genet. 22(1), 82-84. doi: 10.1038/8788

Leduc, M.S., Niu, Z., Bi, W., Zhu, W., Miloslavskaya, I., Chiang, T., et al. (2016). CRIPT exonic deletion and a novel missense mutation in a female with short stature, dysmorphic features, microcephaly, and pigmentary abnormalities. Am J Med Genet A. 170(8), 2206-2211. doi: 10.1002/ajmg.a.37780

Lindor, N.M., Furuichi, Y., Kitao, S., Shimamoto, A., Arndt, C., Jalal, S. (2000). Rothmund-Thomson syndrome due to RECQ4 helicase mutations: report and clinical and molecular comparisons with Bloom syndrome and Werner syndrome. Am J Med Genet. 90(3), 223-228. doi: 10.1002/(sici)1096-8628(20000131)90:3<223::aid-ajmg7>3.0.co;2-z

Piard, J., Aral, B., Vabres, P., Holder-Espinasse, M., Mégarbané, A., Gauthier, S., et al. (2015). Search for ReCQL4 mutations in 39 patients genotyped for suspected Rothmund-Thomson/Baller-Gerold syndromes. Clin Genet. 87(3), 244-251. doi: 10.1111/cge.12361

Powis, Z., Farwell Hagman, K.D., Speare, V., Cain, T., Blanco, K., Mowlavi, L.S., et al. (2018). Exome sequencing in neonates: diagnostic rates, characteristics, and time to diagnosis. Genet Med. 20(11), 1468-1471. doi: 10.1038/gim.2018.11

Reix, P., Derelle, J., Levrey-Hadden, H., Plauchu, H., Bellon, G. (2007). Bronchiectasis in two pediatric patients with Rothmund-Thomson syndrome. Pediatr Int. 49(1), 118-120. doi: 10.1111/j.1442-200X.2007.02292.x

Schwieger-Briel, A., Weibel, L., Sanz, J., Luchsinger, I., Theiler, M. (2021). Poster session at: 14th World Congress of Paediatric Dermatology; 22-25 September 2021, Edinburgh, UK

Siitonen, H.A., Kopra, O., Kääriäinen, H., Haravuori, H., Winter, R.M., Säämänen, A.M., et al. (2003). Molecular defect of RAPADILINO syndrome expands the phenotype spectrum of RECQL diseases. Hum Mol Genet. 12(21), 2837-2844. doi: 10.1093/hmg/ddg306

Siitonen, H.A., Sotkasiira, J., Biervliet, M., Benmansour, A., Capri, Y., Cormier-Daire, V., et al. (2009). The mutation spectrum in RECQL4 diseases. Eur J Hum Genet. 17(2), 151-158. doi: 10.1038/ejhg.2008.154

Shaheen, R., Faqeih, E., Ansari, S., Abdel-Salam, G., Al-Hassnan, Z.N., Al-Shidi, T., et al. (2014). Genomic analysis of primordial dwarfism reveals novel disease genes. Genome Res. 24(2), 291-299. doi: 10.1101/gr.160572.113

Simon, T., Kohlhase, J., Wilhelm, C., Kochanek, M., De Carolis, B., Berthold, F. (2010). Multiple malignant diseases in a patient with Rothmund-Thomson syndrome with RECQL4 mutations: Case report and literature review. Am J Med Genet A. 152A(6), 1575-1579. doi: 10.1002/ajmg.a.33427

Suter, A.A., Itin, P., Heinimann, K., Ahmed, M., Ashraf, T., Fryssira, H., et al. (2016). Rothmund-Thomson Syndrome: novel pathogenic mutations and frequencies of variants in the RECQL4 and USB1 (C16orf57) gene. Mol Genet Genomic Med. 4(3), 359-366. doi: 10.1002/mgg3.209

Sznajer, Y., Siitonen, H.A., Roversi, G., Dangoisse, C., Scaillon, M., Ziereisen, F., et al. (2009). Atypical Rothmund-Thomson syndrome in a patient with compound heterozygous mutations in RECQL4 gene and phenotypic features in RECQL4 syndromes. Eur J Pediatr. 167(2), 175-181. doi: 10.1007/s00431-007-0447-6

Van Maldergem, L., Siitonen, H.A., Jalkh, N., Chouery, E., De Roy, M., Delague, V., et al. (2006). Revisiting the craniosynostosis-radial ray hypoplasia association: Baller-Gerold syndrome caused by mutations in the RECQL4 gene. J Med Genet. 43(2), 148-152. doi: 10.1136/jmg.2005.031781

Wang, L.L., Gannavarapu, A., Kozinetz, C.A., Levy, M.L., Lewis, R.A., Chintagumpala, M.M., et al. (2003). Association between osteosarcoma and deleterious mutations in the RECQL4 gene in Rothmund-Thomson syndrome. J Natl Cancer Inst. 95(9), 669-674. doi: 10.1093/jnci/95.9.669

Wang, T., Chen, L., She, Q., Dong, Y., Deng, Y. (2018). Four novel RECQL4 mutations in four Chinese patients with Rothmund-Thomson syndrome and analysis of RECQL4 mRNA expression level in one typical patient. J Dermatol Sci. 91(3), 335-337. doi: 10.1016/j.jdermsci.2018.06.005

Yadav, S., Thakur, S., Kohlhase, J., Bhari, N., Kabra, M., Gupta, N. (2019). Report of Two Novel Mutations in Indian Patients with Rothmund-Thomson Syndrome. J Pediatr Genet. 8(3), 163-167. doi: 10.1055/s-0039-1684017

Zirn, B., Bernbeck, U., Alt, K., Oeffner, F., Gerhardinger, A., Has, C. (2021). Rothmund-Thomson syndrome type 1 caused by biallelic ANAPC1 gene mutations. Skin Health Dis. 1(1), e12. doi: 10.1002/ski2.12
